# *Cambridge Prisms: Extinction*, after 4 years

**DOI:** 10.1017/ext.2026.10016

**Published:** 2026-06-25

**Authors:** Barry W. Brook, John Alroy

**Affiliations:** 1 School of Natural Sciences, University of Tasmania, Australia; 2 School of Natural Sciences, Macquarie University, Australia

**Keywords:** extinction, extinction accounts, biodiversity loss, impact factor, open access

## Abstract

*Cambridge Prisms: Extinction* was launched to provide an open, synthetic forum for research on the patterns, processes and consequences of extinction. Four years on, the release of the journal’s first impact factor offers a useful moment to look back at what has begun to take shape and to look ahead to what it should now become. The early content shows a young journal whose first visible scholarly impact has been driven largely by synthesis. That was deliberate: a review-led beginning helped define the journal’s intellectual map, draw together conservation biology, palaeontology, modelling, policy, social science and the humanities, and create pathways for more primary and data-driven work. The next stage should build on that foundation through more empirical research and through *Extinction Accounts*, a new article format designed to analyse individual extinctions.

When *Cambridge Prisms: Extinction* began, we argued that extinction research needed an ongoing forum broad enough to connect multidisciplinary research (Brook and Alroy, 2023). Unlike many research areas, it spans deep time and the present, pattern and process, science and the humanities and evidence and action. We later clarified that this scope should be interpreted neither so narrowly that it excludes the field’s transdisciplinary margins, nor so broadly that every study of biodiversity becomes extinction research by default (Alroy and Brook, 2024). The point was to identify a real intellectual nexus. Extinction is an outcome, but it is also a process, an inference problem, a historical event, a policy challenge and often a cultural or ethical question. A journal dedicated to it, therefore, has to be broader than any one discipline, while still asking authors to make extinction itself central to the argument.

Four years is not long in the life of a journal. It is, however, long enough to ask whether a new venue has found a community. In June 2026, *Cambridge Prisms: Extinction* will receive its first Clarivate impact factor of 6.0, a Citescore of 6.8 and a SCImago Journal Rank of 2.5, which collectively placed the journal clearly within the Q1 category for the fields of biodiversity conservation, palaeontology and ecology. But the more important point is already clear: a new open-access journal in a difficult and urgent field has built a visible scholarly footprint quickly enough to be indexed, cited and read as part of the mainstream literature on extinction. In other words, the journal’s central vision was sound.

A snapshot of the journal’s Web of Science record as of June 2026 shows the contents of this first phase. The export contains 67 records: 37 reviews, 23 articles, 6 items classified as editorial material and 1 correction. Those records have accrued 455 all-database citations and 417 Web of Science Core citations. The distribution is characteristic of a new journal, with many recent or specialised papers still uncited and a relatively small number of broad reviews carrying much of the early attention. Reviews make up just over half the corpus but account for most of the citations in the export. This transient imbalance naturally reflects the way the journal was seeded and the role it was designed to play.

The early emphasis on reviews was deliberate. A young journal serving a variety of academic fields first has to make its scope clear. It has to show palaeontologists that contemporary conservation questions belong beside deep-time extinction dynamics, conservation biologists that fossil and historical evidence can inform inference about present risk and scholars in law, economics, ethics, Indigenous studies, language endangerment and cultural analysis that extinction is not solely a biological category. Reviews, perspectives and themed collections gave authors recognisable routes into the journal while establishing a shared map of the subject. They also provided useful anchors for readers coming from outside any one subfield.

The most visible early studies illustrate that breadth. The best-cited syntheses range from late-Quaternary megafaunal losses and their ecological consequences to extinction through the wildlife trade, prediction with species distribution models and the status of the so-called Big Five mass extinctions (Hinsley et al., 2023; Marshall, 2023; Zurell et al., 2023; Svenning et al., 2024). Other highly visible reviews have dealt with phylogenetic diversity, plant extinction at the end of the Cretaceous, morphospace, marine extinction, disease, law, policy, economics, ethics and de-extinction. No single one of these topics defines the journal. Together, they show why extinction research needs a venue organised around mechanism, inference and consequence rather than around a taxon, period or method.

Just as important is the developing body of empirical and data-driven work. For example, individual studies have estimated poorly counted contemporary losses, including Australian non-marine invertebrate extinctions, or asked what historical and contemporary extinction filters can reveal about risk in rails (Lévêque et al., 2024; Woinarski et al., 2024). Others have connected fossil and geochemical evidence to Permian–Triassic shallow-marine extinctions, tested whether phylodynamic models support a pre-impact dinosaur decline or examined how extinction-crisis narratives are understood by conservation actors in the Asian songbird trade (Allen et al., 2024; Fiennes et al., 2024; Foster et al., 2024). Recent articles have also used archives and spatial epidemiology to examine an attempt to induce the extinction of hookworm in Jamaica (Roberts et al., 2025). These articles are not a catalogue of desired subjects. Rather, they show the kind of work that can make the journal more cumulative: studies that measure uncertainty, connect mechanisms to outcomes and make evidence useful beyond the immediate case.

This balance matters because extinction research remains unusually dependent on synthesis. The strongest studies in this field often do more than document loss. They ask how inference can be made from incomplete records, how past events can inform present risks, how population and community processes scale up to irreversible outcomes and how knowledge can be made useful for conservation decisions. The journal should continue to favour work that makes those connections explicit. It should also now attract more primary research, comparative analyses, data papers, methods papers and historically grounded case studies. The impact factor should help with that practical task by giving prospective authors a clearer incentive to send their best work to a specialised journal.

The next stage is also a chance to make extinction research more comparable. One practical step is the development of *Extinction Accounts*, a new article format within the journal’s open-access publishing framework (Cambridge University Press & Assessment, 2026). The purpose is to portray extinction studies as something beyond the mere commemoration of vanished species, ecosystems or cultural forms. Specifically, this format will turn depictions of individual events into evidence-rich accounts of process by asking authors to combine concise biological or historical background with a more substantial account of how extinction unfolded, what evidence supports that interpretation, what uncertainty remains and what lessons can be drawn for present and future risks. In this sense, *Extinction Accounts* are a natural extension of the journal’s mission. They connect narrative clarity with analytical discipline.

We expect these accounts to range widely. Some may focus on individual species, while others may treat families, communities, regional events, ecosystems or cultural extinctions. The useful common denominator is an effort to explain exactly why a reproducing biological, ecological or cultural system ceased to exist, nearly ceased to exist or was declared to have done so. The best accounts will therefore go beyond stories of disappearance, becoming test cases in extinction inference, evidence quality, causation, responsibility and prevention. They should be accessible enough to serve a wide readership, but rigorous enough to be used by researchers, educators, conservation practitioners and policymakers.

We hope the first impact factor will bring new authors and readers to *Cambridge Prisms: Extinction.* More importantly, we hope it will encourage submissions that treat extinction as a problem requiring integration: of archives and genomes, fossils and monitoring data, models and mechanisms, ethics and governance. The journal was created to make that integration easier. Its first 4 years suggest that the need for such a forum has only grown.

## Data Availability

Not applicable.
