# Supplementary material for: Cambridge Prisms: Extinction, after 4 years
Source: Camb Prism Extinct. 2026 Jun 25;4:e10. doi: 10.1017/ext.2026.10016 (PMC13438210; doi:10.1017/ext.2026.10016)
Supplement: Peer review history

# Editor report: Recommendation: *Cambridge Prisms: Extinction*, after 4 years — R0/PR2

**DOI:** 10.1017/ext.2026.10016.pr2

**Affiliations:** Cambridge University Press & Assessment, United Kingdom of Great Britain and Northern Ireland

Accepted

# Editor report: Decision: *Cambridge Prisms: Extinction*, after 4 years — R0/PR3

**DOI:** 10.1017/ext.2026.10016.pr3

**Affiliations:** Cambridge University Press & Assessment, United Kingdom of Great Britain and Northern Ireland

No accompanying comment.
